# Response to “Occupational physical behavior in eldercare workers: moving beyond description toward health implications”

**DOI:** 10.1093/annweh/wxag018

**Published:** 2026-03-31

**Authors:** Nestor Lögdal, Svend Erik Mathiassen, Jennie A Jackson, David M Hallman

**Affiliations:** Department of Occupational Health, Psychology and Sports Sciences, University of Gävle, Kungsbäcksvägen 47, 801 76 Gävle, Sweden; Department of Occupational Health, Psychology and Sports Sciences, University of Gävle, Kungsbäcksvägen 47, 801 76 Gävle, Sweden; Department of Occupational Health, Psychology and Sports Sciences, University of Gävle, Kungsbäcksvägen 47, 801 76 Gävle, Sweden; Department of Occupational Health, Psychology and Sports Sciences, University of Gävle, Kungsbäcksvägen 47, 801 76 Gävle, Sweden

We appreciate the constructive letter to the editor from Amalia et al. We are pleased that our study has stimulated engagement and discussion.

We agree that predicting meaningful clinical outcomes from descriptive physical behavior patterns remains a key challenge, and that longitudinal research is needed to clarify whether specific temporal patterns of time on feet and sitting influence recovery, musculoskeletal pain, sickness absence, and long-term work sustainability. In that context we share the view of Amalia et al. that prospective designs associating behavioral metrics with health outcomes are a necessary next step. At the same time, we would like to emphasize that our study was framed within the substantial evidence base from other occupations indicating that prolonged time on feet and extended standing are associated with adverse health outcomes (Waters and Dick 2015; Coenen et al. 2017, 2018; Øverås et al. 2020; Ahmadi et al. 2024). We believe that there is little reason that eldercare workers would be exempt from the risks observed in other similarly exposed populations. Thus, our intention was to provide a detailed and methodologically robust characterization of temporal exposure patterns in eldercare, serving as an empirical foundation for subsequent outcome-oriented research in this specific workforce.

We welcome the suggestion by Amalia et al. to integrate physiological indicators, such as heart rate variability (HRV) and biomarkers of exertion. Such measures may substantially strengthen mechanistic understanding of how occupational physical behaviors relate to strain, recovery, and, eventually, disorders. As we have argued elsewhere (Lögdal et al. 2025), there is a need for integrative physiological studies combining objective cardiovascular measures, such as heart rate, HRV, and oxygen uptake, with biomechanical data, such as local loads on the lower back, shoulders, and arms, and subjective measures, such as perceived demands. Experimental or observational studies integrating physiological monitoring, biomechanical assessment, and contextual factors, including psychosocial demands, could clarify how temporal exposure patterns translate into short- and long-term health outcomes, and provide a more complete picture of exposure and response. This is a direction we are currently pursuing in our own research.

We also agree that interpretation of results must remain grounded in the organizational and systemic context. Our study considers Swedish eldercare, with its specific staffing structures, scheduling systems, and division of labor. As discussed in the manuscript, such contextual factors likely influence the distribution of tasks in the workforce, and thus opportunities for sitting and interruptions of time on feet. We encourage replications of our study in other organizational systems and national contexts to determine the extent to which the observed temporal patterns represent general characteristics of eldercare work or context-specific features.

In summary, we agree that future research should move toward longitudinal designs, integrate physiological indicators, and remain sensitive to the organizational context. We also see value in complementing time-based behavioral metrics, both at the task level and across the whole job, with objective assessments of biomechanical loads and postural demands, so that co-occurring aspects of occupational exposure are addressed in parallel rather than as isolated factors.

## Data Availability

No data were used in this study.
